# Panic Disorder Severity Scale self-report: transcultural validation and sensitivity to change of the French-Canadian adaptation

**DOI:** 10.1192/j.eurpsy.2022.982

**Published:** 2022-09-01

**Authors:** P. Roberge, M. Provencher, P. Norton, N. Carrier, P. Marx, J. Couture, A. Benoît

**Affiliations:** 1 Université de Sherbrooke, Médecine De Famille Et Médecine D’urgence, Sherbrooke, Canada; 2 Université Laval, École De Psychologie, Québec, Canada; 3 Cairnmillar Institute, Psychology, Hawthorn East, Australia

**Keywords:** Psychometric properties, panic disorder, French validation, Assessment scale

## Abstract

**Introduction:**

The self-report version of the Panic Disorder Severity Scale (PDSS-SR) is a reliable and valid instrument to assess panic disorder, but is unavailable in French.

**Objectives:**

The aim of this study was to conduct a transcultural validation of the French-Canadian PDSS-SR and examine its psychometric properties.

**Methods:**

This study is part of a pragmatic RCT of group transdiagnostic CBT for anxiety disorders, and includes 272 adults meeting DSM-5 panic disorder diagnostic criteria. At baseline, participants completed the Anxiety and Related Disorders Interview Schedule (ADIS-5), the French-Canadian PDSS-SR and self-report measures. Convergent validity was assessed with Spearman correlations, Cronbach’s α was used to analyse internal consistency, and confirmatory factor analysis (CFA) evaluated its factor structure. Sensitivity to change was assessed with paired sample t-tests in patients (n = 72) meeting DSM-5 criteria for panic disorder at baseline with posttreatment data.

**Results:**

108 patients met DSM-5 criteria for panic disorder, including 58 with agoraphobia. The majority were women (85.3%) and mean age was 37.1 (SD = 12.4). Internal consistency (Cronbach’s α) was 0.91. For convergent validity, the highest correlation was with the Beck Anxiety Inventory (r = 0.64). CFA suggested a two-factor model. Optimal threshold for probable diagnosis was 10. Analyses support sensitivity to change when comparing transdiagnostic group CBT and control conditions.

**Conclusions:**

With its good psychometric properties in primary care patients, the French-Canadian self-report version of the Panic Disorder Severity Scale is an efficient and practical instrument for both clinicians and researchers working in the field of mental health.

**Disclosure:**

No significant relationships.

